# Novel probe-based melting curve assays for the characterization of fluoroquinolone resistance in *Mycoplasma genitalium*

**DOI:** 10.1093/jac/dkac097

**Published:** 2022-03-30

**Authors:** Jacob A. Tickner, Catriona S. Bradshaw, Gerald L. Murray, David M. Whiley, Emma L. Sweeney

**Affiliations:** 1 The University of Queensland Centre for Clinical Research (UQ-CCR), Faculty of Medicine, The University of Queensland, Brisbane, Queensland, Australia; 2 Melbourne Sexual Health Centre, Alfred Hospital and Central Clinical School, Monash University, Melbourne, Victoria, Australia; 3 Central Clinical School, Monash University, Melbourne, Victoria, Australia; 4 The Department of Obstetrics and Gynaecology, University of Melbourne, Parkville, Victoria, Australia; 5 Centre for Women’s Infectious Diseases, The Royal Women’s Hospital, Parkville, Victoria, Australia; 6 Molecular Microbiology Research Group, Murdoch Children’s Research Institute, Parkville, Victoria, Australia; 7 Pathology Queensland Central Laboratory, Queensland, Australia

## Abstract

**Background:**

*Mycoplasma genitalium* infection is a sexually transmitted infection that has rapidly become resistant to mainstay treatments. While individualized treatment approaches have been recommended and adopted for macrolides, individualized therapy for fluoroquinolones has not yet been explored, due to a lack of commercial molecular assays and a lack of confidence in specific mutations associated with resistance. In another recent study, we defined a clear role and diagnostic utility in focusing on the absence of resistance mutations to inform microbial cure with fluoroquinolone antimicrobials.

**Methods:**

We developed two proof-of-concept molecular tests that focus on detection of *M. genitalium* and characterization of WT *parC* sequences that are strongly linked to fluoroquinolone susceptibility.

**Results:**

We screened a total of 227 *M. genitalium*-positive samples using novel molecular beacon and dual hybridization probe assays. These assays were able to detect *M. genitalium* and characterize fluoroquinolone susceptibility in 143/227 (63%) samples, based on clear differences in melting peak temperatures. The results of these molecular assays were in 100% agreement with ‘gold standard’ Sanger sequencing. Additionally, WT *parC* sequences were readily distinguished from *M. genitalium* samples harbouring *parC* mutations of known or suspected clinical significance. The ability of the assays to successfully characterize fluoroquinolone susceptibility and resistance was reduced in low *M. genitalium* load samples.

**Conclusions:**

These proof-of-concept assays have considerable potential to improve individualized treatment approaches and rationalize tests of cure for *M. genitalium* infection. The ability to initiate individualized treatment in up to two-thirds of cases will enhance antimicrobial stewardship for this challenging pathogen.

## Introduction

*Mycoplasma genitalium* sexually transmitted infection (STI) is associated with acute and chronic urethritis in male individuals, and cervicitis and pelvic inflammatory disease in female individuals.^1^ It is now also recognised as an antimicrobial-resistant (AMR) ‘superbug’. In 2019, the US CDC escalated *M. genitalium* to the AMR threats ‘watch list’ due to the fact that it has rapidly become resistant to recommended treatments, including macrolides and fluoroquinolones.^2–5^ In urban settings worldwide, rates of macrolide resistance typically exceed 50%. Despite fluoroquinolone resistance typically being less common, the rates are nevertheless on the rise globally, and fluoroquinolone clinical treatment failures and elevated MICs due to mutations in the *M. genitalium parC* gene are increasingly being reported.^2–4,6^ In the Asia-Pacific region, rates of fluoroquinolone resistance mutations are among the highest globally, at 14.3% according to recent meta-analyses,^3^ and a recent study in China reported alarmingly high fluoroquinolone resistance levels at ∼77%.^7^

Such high levels of *M. genitalium* AMR have necessitated the development and use of molecular tests to detect resistance and directly inform selection of antimicrobials. This first generation of PCR-based resistance assays are available for detection of the well-characterized *M. genitalium* 23S rRNA mutations that confer macrolide resistance.^8–10^ This approach has resulted in a significant improvement in first-line cure and is now suggested in STI treatment guidelines of many countries including Australia, the USA and the UK.^11–13^ With rising rates of AMR, resistance-guided therapy approaches that detect fluoroquinolone resistance markers also appear to be warranted; however, this approach is more complex than for macrolides. This is because unlike macrolide resistance, which involves mutations in only one codon of the 23S rRNA gene, there are numerous potential sequence targets to consider for fluoroquinolone resistance, including various mutations in *parC*, *parE*, *gyrA* and *gyrB.*^2–4,6,14–16^ There is now increasing clarity on the respective roles of these genes in conferring fluoroquinolone resistance in *M. genitalium*. For instance, it is now clear that mutations in the *parC* gene of *M. genitalium* are the most common, and appear to be better predictors of treatment failure with moxifloxacin and sitafloxacin, compared with mutations occurring in *gyrA* and other genes.^2,3,6^ Also, both clinical treatment failure and MICs are typically higher for strains with concurrent *parC* and *gyrA* mutations.^14^ However, controversy remains as to the relative contribution of specific mutations, including those within *parC*, and notably which mutations should be included in assays to most effectively guide fluoroquinolone treatment.^17^

For ParC, there are two key amino acids at positions 83 (*M. genitalium* numbering, serine; nucleotide G248) and 87 (aspartic acid; nucleotide G259) that impact upon the efficacy of moxifloxacin and to a lesser degree sitafloxacin. Of the recognized alterations, there is strong evidence indicating a role of the ParC S83I substitution (caused by the SNP G248T), which leads to clinical failure in approximately two-thirds of cases,^2,6^ whereas questions remain over the significance of other rarer changes at S83 (e.g. S83C/R/N) and D87 (e.g. D87H/Y/N/G).^14,17,18^

Evidence supports the inclusion of the S83I mutation in a molecular test for *M. genitalium* fluoroquinolone resistance, but it is less certain whether there is a role for the inclusion of S83C, S83R, S83N, D87N, D87Y and D87H due to the fact that they are relatively scarce, particularly in the Asia-Pacific region,^2–4,6,19^ and less commonly associated with treatment failure. This presents a dilemma for risk-averse commercial assay developers, who appear to have avoided *M. genitalium* fluoroquinolone resistance due a lack of certainty on the contribution of these markers to treatment failure and concerns over assay complexity. When considering these issues in a recent opinion piece^20^ we provided compelling evidence based on local Australian data to show that reliance on the S83 codon could indeed be used to theoretically achieve ∼97% cure rates in infected individuals treated with moxifloxacin. Thus, at least in our population, high treatment success can be achieved by identifying the S83 WT status in an infection, and potentially disregarding mutations at codon 87 of ParC. A novel aspect of our approach was the focus on ensuring detection of the *WT* sequence (as opposed to specific resistance mutations). This strategy prioritizes detection of a susceptible infection, rather than trying to infer susceptibility in the absence of detecting a mutation. We believe this approach could also be used to offer a simple solution to address the codon 87 issues discussed above, where these mutations are uncommon and their contribution to treatment failure is less clear. Here we have developed and validated novel proof-of-concept assays that target *both* the S83 and D87 codons of ParC, as a means of using the WT sequence at both positions in a resistance-guided strategy. This ‘WT’ approach would promote antimicrobial stewardship, achieving high first-line cure following moxifloxacin treatment in susceptible infections.

## Methods

### Overview

**Figure 1.**
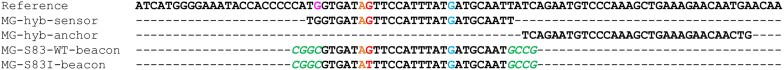
Overview of MGfl-MolBeac-PCR (molecular beacon) and MGfl-HYB-PCR (dual hybridization probe) designs. Both probe designs encompass the *parC* nucleotides 247 (orange), 248 (red) and 259 (blue) that predict fluoroquinolone susceptibility and resistance. The dual hybridization probe also includes the 241 (magenta) nucleotide, which encodes the exceedingly rare G81C mutation, which has questionable clinical significance with respect to fluoroquinolone resistance. *M. genitalium parC* WT sequence is shown at the top of the figure, with all designed probes shown beneath, noting that all probes, with the exception of the MG-S83I-beacon, are designed to match the *WT* sequence. Molecular beacon stem sequences are shown in green italics, noting that they are different from the reference sequence. This figure appears in colour in the online version of *JAC* and in black and white in the print version of *JAC*.

Two PCR assays (MGfl-HYB-PCR and MGfl-MolBeac-PCR) were developed, both of which used post-PCR melting curve analysis to characterize the 83 and 87 codons (Figure 1). The MGfl-HYB-PCR assay used a dual hybridization probe format, whereas the MGfl-MolBeac-PCR assay utilized molecular beacons. The key design feature was that both assays included a probe spanning both the 83 and 87 codons with 100% match to the WT sequences. Hence, any mismatches to this WT probe (e.g. mutations at either S83 or D87) would result in a lower melting temperature during melting curve analysis. Note that the MGfl-MolBeac-PCR assay also included a separate specific probe for the S83I mutation. Both assays were applied to routinely collected *M. genitalium*-positive samples, and the results were compared with ‘gold standard’ *parC* Sanger sequencing.^4^

### MGfl-HYB-PCR (dual hybridization probe) assay

The MGfl-HYB-PCR assay utilized two primers (MG-*parC*-F and MG-*parC*-R; Table 1; this study) and a single set of hybridization probes (MG-hyb-sensor and MG-hyb-anchor; Table 1; this study). The Roche Light Cycler 480 Genotyping Master Mix served as the basis for this assay, and each reaction contained 10 pmol of forward primer, 30 pmol of reverse primer, 4 pmol each of the sensor and anchor hybridization probes and 2.0 μL of nucleic acid extract, in a total reaction volume of 20 μL. Reactions were cycled using the Rotor-Gene Q (QIAGEN, Australia) real-time PCR instrument, using an initial hold at 95°C for 10 min, followed by 55 cycles of 95°C for 10 s, 55°C for 20 s (acquisition on red channel; excitation/emission of 625/660 nm) and 72°C for 30 s, followed by a standard melt programme of 40°C–95°C, increasing by 1°C per step, with a 5 s hold for each step. For the MGfl-HYB-PCR, we only assigned results as either WT or mutant. In doing so, the expectation was that only WT sequences would provide the maximum melt peak temperature, whereas any mutation in the sensor probe region would lead to a decreased melting peak temperature and indicate the presence of a *parC* mutation.

**Table 1.** Primers and probes used in the MGfl-HYB-PCR (hybridization probe) and MGfl-MolBeac-PCR (molecular beacon) assays

Name	Sequence (5′→3′)	Assay
MG-*parC*-F	TCAAATGGGCTTAAAACCCACCACT	MGfl-HYB-PCR & MGfl-MolBeac-PCR
MG-*parC*-R	CTTAAGCGGGTTTCTGTGTAACGCAT	MGfl-HYB-PCR & MGfl-MolBeac-PCR
MG-hyb-sensor probe	TGGTGATAGTTCCATTTATGATGCAATT-FAM	MGfl-HYB-PCR (sensor probe)
MG-hyb-anchor probe	Cy5-TCAGAATGTCCCAAAGCTGAAAGAACAACTG-Phos	MGfl-HYB-PCR (anchor probe)
MG-S83-WT-beacon probe	FAM-CGGCGTGATAGTTCCATTTATGATGCAATGCCG-IAbFQ	MGfl-MolBeac-PCR (WT molecular beacon)
MG-S83I-beacon probe	Cy5- CGGCGTGATATTTCCATTTATGATGCAATGCCG-IAbRQ	MGfl-MolBeac-PCR (S83I molecular beacon)

### MGfl-MolBeac-PCR (molecular beacon) assay

The MGfl-MolBeac-PCR assay utilized the same previously described *parC* primers (MG-*parC*-F and MG-*parC*-R; Table 1) but incorporated two molecular beacon probes: one probe for the WT sequence (MG-S83-WT-beacon; Table 1; this study) and a second probe specific to the S83I (G248T) mutation (MG-S83I-beacon; Table 1; this study). Note that signal from each probe was distinguished via the use of different fluorophores and associated detection channels. The Roche Light Cycler 480 Genotyping Master Mix was again used as the basis for our molecular beacon assay. Each reaction contained 3 pmol of forward primer, 30 pmol of reverse primer, 4 pmol each of MG-S83-WT and MG-S83I molecular beacons and 2.0 μL of nucleic acid extract in a total reaction volume of 20 μL. Reactions were cycled using the Rotor-Gene Q real-time PCR instrument, using an initial hold at 95°C for 10 min, followed by 55 cycles of 95°C for 10 s, 55°C for 20 s (data acquisition on green and red channels; excitation/emission of 470/510 and 625/660 nm, respectively) and 72°C for 30 s, followed by a modified standard melt programme of 40°C–65°C, increasing by 1°C per step, with a 30 s hold for each step. It should be noted that for the MGfl-MolBeac-PCR assay a small number of *M. genitalium*-negative samples exhibited very minor background fluorescence and so we established specific melt range criteria for our analysis and only assigned results as either ‘WT’ or ‘mutant’ for the WT probe melting curves, and as either ‘S83I’ or ‘not-S83I’ on the basis of the S83I probe melting curves; these results were then combined to provide a final result call. In doing so, again the expectation was that WT sequences would provide the maximum melting temperature for the WT probe, and thus any mutation in the probe target would lead to a decreased melting temperature. Likewise, only S83I mutants were expected to provide the maximum melting temperature for the S83I probe. This approach of only relying on the maximum temperature, and not trying to distinguish between any of the lower melting curves, was done to ensure the greatest confidence in result calling.

### Sample bank

A bank of 242 *M. genitalium*-positive samples were used in this study. These were from routine *M. genitalium* PCR screening collected between 2020 and 2021 in Queensland, Australia and were kindly provided by our local pathology provider. The samples had previously been DNA-extracted using the Roche Cobas 4800 (*n *= 125) or MagNA Pure 96 (*n *= 117) automated nucleic acid extraction platforms, had tested positive for *M. genitalium* at Pathology Queensland using an MgPa-PCR,^21^ and were subsequently screened for the presence of fluoroquinolone resistance mutations by *parC* Sanger sequencing as part of an ongoing research study.^4^ Specimen extracts included urine (*n *= 126), female genital swabs (*n *= 90), male genital swabs (*n *= 9), anorectal swabs (*n *= 9) and ‘specimen with no site specified’ (*n *= 8) obtained from 131 male patients and 111 female patients. A bank of known *M. genitalium*-negative patient samples (*n *= 60) and a panel of nucleic acid extracts from additional organisms including pathogens and species (*n *= 23) commonly found at urogenital sites (*Acinetobacter baumannii*, *Bacillus cereus*, *Bacillus subtilis*, *Candida albicans*, *Candida tropicalis*, *Citrobacter freundii*, *Chlamydia trachomatis*, *Enterococcus faecium*, *Escherichia coli*, *Gardnerella vaginalis*, *Micrococcus luteus*, *Mycoplasma hominis*, *Neisseria subflava*, *Neisseria gonorrhoeae*, *Proteus mirabilis*, *Pseudomonas aeruginosa*, *Staphylococcus aureus*, *Staphylococcus epidermidis*, *Streptococcus pyogenes*, *Streptococcus agalactiae*, *Ureaplasma parvum*, *Ureaplasma urealyticum* and *Vibrio parahaemolyticus*) were also screened in order to assess the specificity of the assays. Given sample extracts had been stored at −20°C for up to 2 years, all samples were retested using the MgPa-PCR assay as part of this study to ensure amplifiable *M. genitalium* was still present in the appropriate samples. Ethics approval for this study was provided by the Children’s Health Queensland Human Research Ethics Committee (HREC/12/QRCH/139).

### Limit of detection

Pooled nucleic acid extracts from urine of *M. genitalium*-negative patients were spiked with a commercially available quantified positive control (AmpliRun^®^*Mycoplasma genitalium* DNA control, Vircell). Dilutions (1, 2.5, 5, 10 and 100 genome copies per reaction) were then tested in triplicate on three occasions, in order to determine the limit of detection for both assays.

### Statistical analyses

The MgPa-PCR cycle threshold (Ct) values of samples were grouped according to whether the sample was successfully or unsuccessfully characterized using both the molecular beacon and dual hybridization probe assays. Grouped values were then assessed for normality using D’Agostino–Pearson testing and QQ-plot visualization. The data were deemed normally distributed and assessed using parametric unpaired *t*-test. Statistical analyses were performed using GraphPad Prism 9.2.0.

## Results

**Figure 2.**
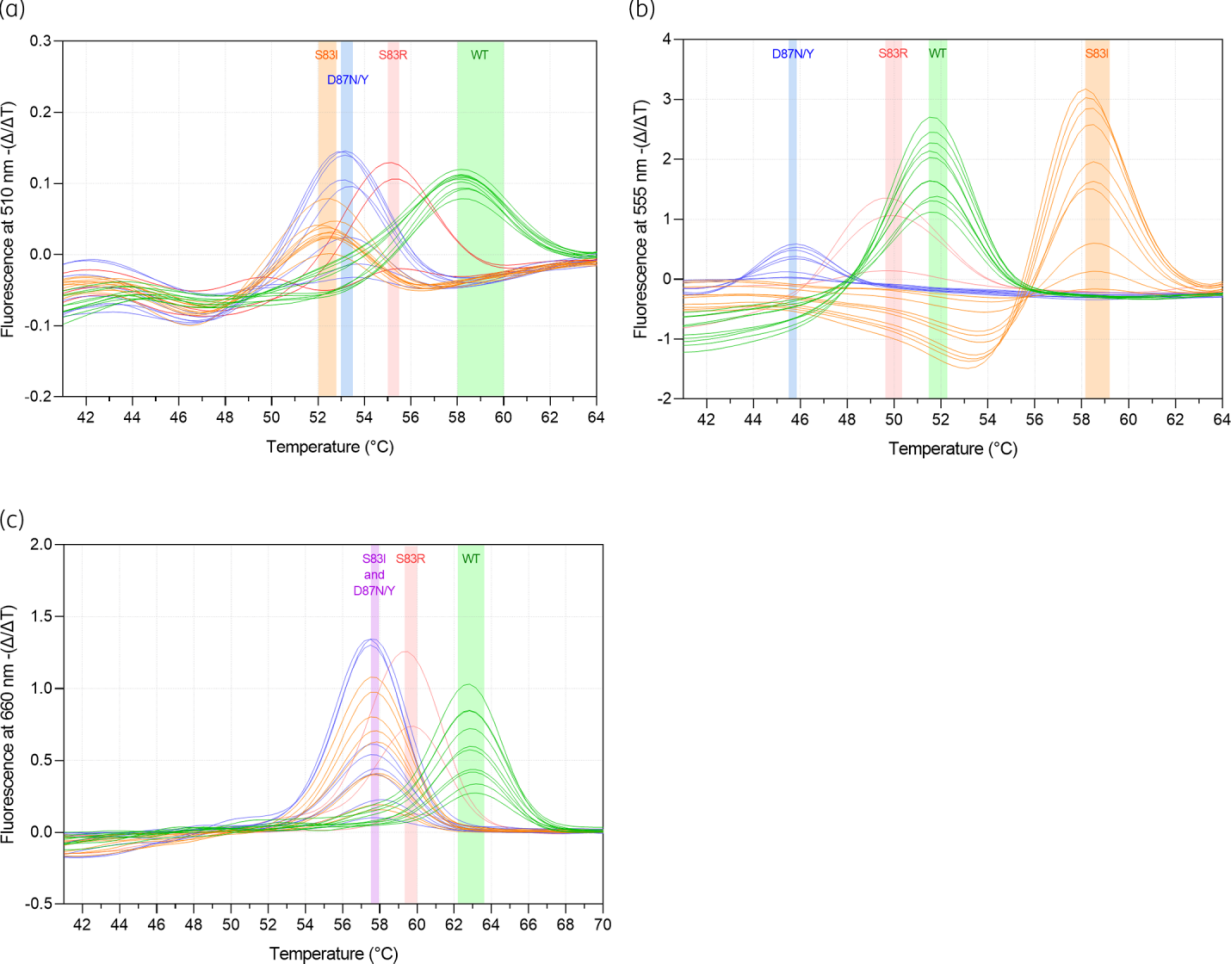
Representative melting peaks for ParC WT (green) and ParC-S83I fluoroquinolone resistance mutation in molecular beacon assays (a and b) and dual hybridization probe assays (c). This figure appears in colour in the online version of *JAC* and in black and white in the print version of *JAC*.

Results are summarized in Table 2 and Figure 2, and all individual sample results are also detailed in Table S1, available as Supplementary data at *JAC* Online. When retested via the MgPa-PCR, 94% (227/242) of samples provided positive results, indicating that the DNA for the remaining 6% of samples (*n *= 15) may have degraded or they were otherwise beyond the threshold of sensitivity of the MgPa-PCR. These samples were excluded from the study, but are presented and highlighted in red within Table S1 for the purposes of transparency.

**Table 2.** Summary of 227 results across the two assays

Sanger sequencing	Molecular beacon assay					Dual hybridization probe assay			
	MGfl-MolBeac-WT melt (°C) (mean)	MGfl-MolBeac-WT call	MGfl-MolBeac-S83I melt (°C) (mean)	MGfl-MolBeac-S83I call	MGfl-MolBeac final call	MGfl-HYB melt (°C) (mean)	MGfl-HYB final call	MgPa PCR Ct (mean)	No. of samples
WT	58–59.5 (58.6)	WT	51.5–52.2 (51.8)	not-S83I	WT	62.2–63.5 (63)	WT	26–42 (32)	82
	n/a	no call	51.5–51.7 (51.6)	not-S83I	not S83I	63–63.3 (63.1)	WT	35, 39	2
	58.7–60	WT	51.7–52	not-S83I	WT	n/a	no call	34, 35, 39	3
	n/a	no call	n/a	no call	no call	63–63.7 (63.3)	WT	32–40 (37)	14
	n/a	no call	n/a	no call	no call	n/a	no call	31–40 (37)	42
S83I mutant	52–52.8 (52.5)	mutant	58.2–58.8 (58.5)	S83I mutant	S83I mutant	57.5–58 (57.7)	mutant	28–39 (32)	14
	52.2, 52.5	mutant	n/a	no call	mutant	n/a	no call	31, 32	2
	52.5	mutant	58.3	S83I mutant	S83I mutant	n/a	no call	33	1
	n/a	no call	58.5–59.2 (58.8)	S83I mutant	S83I mutant	56.7–58 (57.7)	mutant	30–39 (34)	16
	n/a	no call	58.5–59.2 (58.8)	S83I mutant	S83I mutant	n/a	no call	32–39 (36)	10
	n/a	no call	n/a	no call	no call	57.7, 58	mutant	34, 37	2
	n/a	no call	n/a	no call	no call	n/a	no call	33–41 (38)	24
D87N mutant	53–53.5 (53.2)	mutant	45.5–45.8 (45.7)	not-S83I	mutant, not S83I	57.5–57.8 (57.6)	mutant	27–35 (30)	6
	n/a	no call	n/a	no call	no call	n/a	no call	37	1
D87Y mutant	53.3, 53.3	mutant	45.5, 45.5	not-S83I	mutant, not S83I	57.7, 58	mutant	36, 37	2
S83R mutant	55.5	mutant	49.8–50.3 (50)	not-S83I	mutant, not S83I	59.7–59.8 (59.8)	mutant	31–37 (34)	3
	n/a	no call	n/a	no call	no call	n/a	no call	39	1
G81C mutant	n/a	no call	n/a	no call	no call	n/a	no call	38, 38	2

No call, no call was able to be determined due to the lack of an evaluable melting peak; n/a, not available; MGfl-MolBeac-WT, molecular beacon, WT probe assay; MGfl-MolBeac-S83I, molecular beacon, S83I mutant probe assay; MGfl-MolBeac final call, combined results from the WT and S83I mutant molecular beacon assays; MGfl-HYB melt, dual hybridization probe assay (this assay contains probes with 100% similarity to WT).

**Figure 3.**
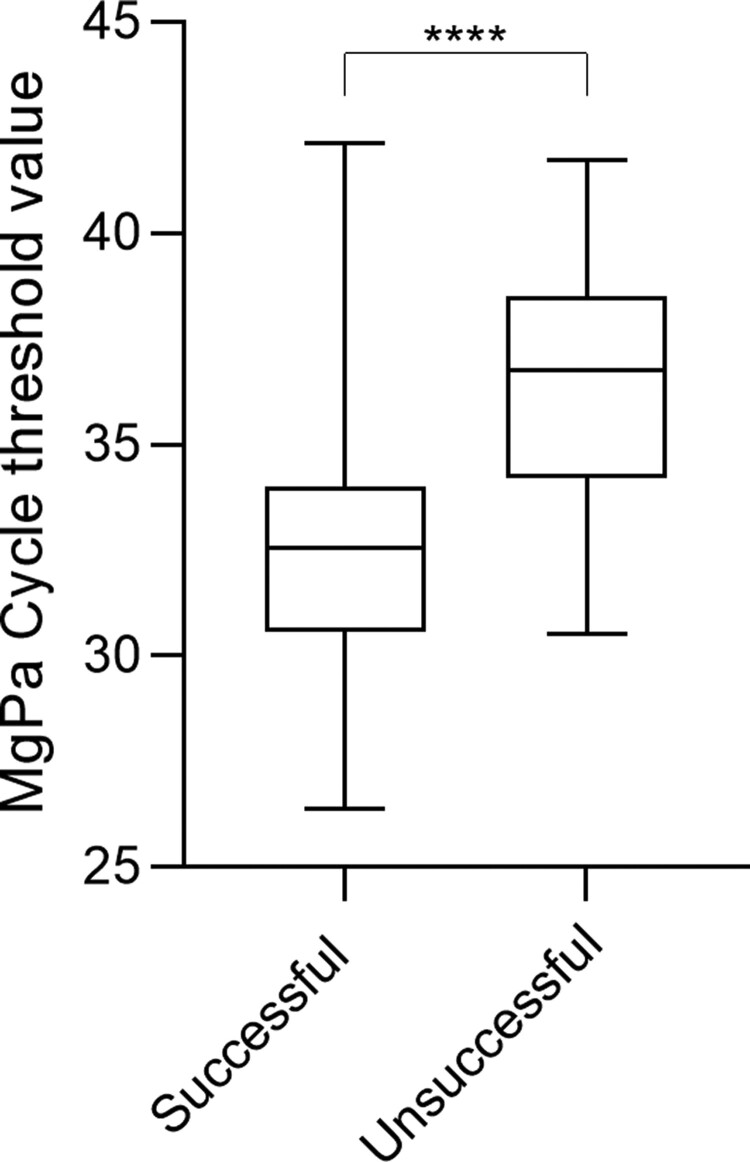
Boxplot of MgPa-PCR Ct values of samples that were successfully or unsuccessfully characterized across the molecular beacon and dual hybridization probe assays. Parametric analysis via unpaired *t*-test was performed and this association was found to be statistically significant (****P < 0.05).

The MGfl-MolBeac-PCR was able to characterize 143/227 (63%) samples, and typed these as WT (*n *= 86), S83I (*n *= 41), mutant/not-S83I (*n *= 11), not-S83I (*n *= 3) and mutant (*n *= 2) (Table 2). All of these results were consistent with the results of Sanger sequencing. The remaining 84 samples did not provide any fluorescent signal above the evaluable threshold for either of the MGfl-MolBeac-PCR probes (Table 2 and Figure 2a and b). Though no MgPa Ct value could be determined as a cut-off for success using either the MGfl-MolBeac-PCR or MGfl-HYB-PCR assay, analysis of successful and unsuccessful tests compared with MgPa testing showed unsuccessful samples had a statistically higher mean MgPa Ct of 36, when compared with successfully tested samples (mean MgPa Ct = 32; Figure 3). There was no difference in the ability of our assays to detect *M. genitalium* based on the nucleic acid extraction methods used within this study (Table S1).

The MGfl-HYB-PCR was also able to characterize 143/227 (63%) samples as WT (*n *= 100) or mutant (*n *= 43), which was again consistent with the results of Sanger sequencing (note that these samples comprised 127/143 of the samples successfully characterized by the MGfl-MolBeac-PCR). Similar to the MGfl-MolBeac-PCR, a total of 84 samples did not provide any fluorescent signal in the MGfl-HYB-PCR and therefore were unable to be characterized (Table 2 and Figure 2c).

None of the *M. genitalium*-negative samples (*n *= 60), nor the bank of other STI-causing and genital microorganisms (*n *= 23), provided any specific signal in either assay, thus confirming their specificity. Using pooled nucleic acid extract from urine of *M. genitalium*-negative patients, spiked with commercially available quantitated positive control (Vircell AmpliRun), both assays consistently detected down to 2.5 genome copies per reaction in all replicate reactions, while at lower dilutions, target amplification was not consistent across all replicate reactions.

Comparison of the MGfl-HYB-PCR and MGfl-MolBeac-PCR probe sequences with the *parC* Sanger sequence from the local samples tested within the study also revealed a potentially problematic proximal mutation at codon 81 in two samples. This G81C mutation fell within the probe region of the MGfl-HYB-PCR sensor probe, but was outside of the MGfl-MolBeac-PCR sequence targets (see Figure 1). G81C (and other potential mutations at the G81 codon, such as G81S) were therefore predicted to impact upon the MGfl-HYB-PCR melting curve analysis but not the MGfl-MolBeac-PCR. Nevertheless, both samples failed to be characterized in both the MGfl-HYB-PCR and MGfl-MolBeac-PCR assays, presumably due to the low *M. genitalium* loads (Ct values of 37 and 38 in the MgPa-PCR, Table S1) that were below the detection limit of the genotyping assays.

## Discussion

Fluoroquinolone treatment failures are becoming a very challenging issue for clinicians in the management of *M. genitalium* infections. In urban centres in Australia, approximately 30% of macrolide-resistant infections have relevant quinolone resistance mutations.^19^ Clinicians are increasingly recognizing the need to incorporate the detection of fluoroquinolone resistance markers in testing and treatment strategies to improve first-line cure and antimicrobial stewardship.^20^ However, until recently the lack of evidence for the contribution of specific mutations to treatment failure, scarcity of commercial molecular tests to detect *M. genitalium parC* mutations,^22–24^ and the lack of markers for the detection of fluoroquinolone susceptibility have slowed efforts to adopt these approaches. We previously published a TaqMan-based PCR method that can distinguish WT S83 from S83I,^25^ but that assay failed to consider the D87 alterations. In this proof-of-concept study, we successfully explored two novel simplistic PCR designs, utilizing probes spanning both the ParC S83 and D87 residues, to simultaneously distinguish WT from mutant at both amino acids.

A key benefit of our approach, in addition to simplicity, is that it does not rely on the *absence* of a resistance mutation to infer susceptibility; rather it focuses on detection of the WT sequence to determine susceptibility with more confidence. The key design feature is that the probes matched 100% to the *parC* WT sequence (with the exception of the S83I molecular beacon). This is important in terms of melting curve analysis (summarized in Table 2 and in detail in Table S1) because the melting temperatures of probe-based melting curve assays are inherently linked to the number of matching bases; the highest melting temperature is achieved where all bases within the probe are matching (in this case for WT) and any mismatches can only result in a decrease in melting temperature, not an increase. As such, a maximum melting temperature should theoretically predict WT with very high accuracy, and in fact is shown here with the same accuracy as DNA sequencing. In turn, this ability to accurately assign WT status provides greater confidence for clinicians in the selection of a fluoroquinolone such as moxifloxacin for treatment.

Overall, both the MGfl-HYB-PCR and MGfl-MolBeac-PCR assays were 100% specific for the detection of *M. genitalium* fluoroquinolone susceptibility (ParC WT) and were able to easily discriminate ParC WT from other common ParC mutations based on melting peak temperatures (the closest WT and mutant peaks for either assay differed by approximately 2°C–3°C). In addition, the MGfl-MolBeac-PCR assay (through inclusion of the MG-S83I-beacon probe) was also able to confidently predict the presence of the ParC-S83I mutation. From a clinical perspective, the inclusion of an assay that can detect both ParC WT and S83I, with macrolide-resistance testing at diagnosis could provide various options for clinical management, and enable clinicians to confidently select appropriate first-line antimicrobials and also rationalize test of cure for patients who may be more at risk of treatment failure. For example, in cases where a patient is screened and the infection is macrolide resistant but ParC WT sequences are identified, the patient could be treated with moxifloxacin first line, and based on our current data, which indicate that WT is associated with ≥97% cure rates,^20^ a test of cure could be limited to patients with persistent symptoms. Whereas, in patients with a macrolide-resistant infection harbouring the ParC-S83I (G248T) mutation, ideally the clinician would choose a non-quinolone option, such as minocycline or pristinamycin, both of which achieve cure rates in the order of 70%–75%.^26,27^ If the only option available is moxifloxacin, based on our current data we would anticipate rates of cure around 50%–60%.^2,19,20^ In this case, a test of cure following treatment would obviously be routinely recommended. As the prevalence of other ParC mutations (S83R, S83N and D87N, D87H, D87Y) is low, more data are needed before we can be confident about their contribution to moxifloxacin failure and ultimately their role in diagnostic assays. It should also be noted that where samples failed to be characterized by these ParC melting peak assays, then it is best to estimate likelihood of resistance for the patient based on (i) their prior treatment history for the infection; and (ii) local fluoroquinolone resistance levels. If the likelihood of fluoroquinolone resistance is high, then cure is more likely to be achieved with an agent such as pristinamycin or minocycline, where available. If fluoroquinolone resistance is low in the population, then moxifloxacin is likely to be effective. A discussion with patients around the pros and cons of proceeding with fluoroquinolones in the absence of resistance data is also an essential part of reaching an informed decision.

There are some limitations of this study. Firstly, the sensitivity of both assays was limited, with only 159/227 (70%) of *M. genitalium* samples yielding evaluable melt profiles of sufficient quality for interpretation. Given the majority of these samples contained sufficient *M. genitalium* DNA to amplify by quantitative PCR, additional optimization of these assays is needed and will improve the overall performance of the assay. Nevertheless, our results highlight a timely and important proof-of-concept application for these fluorescent probes for characterization of ParC fluoroquinolone susceptibility and resistance. Additionally, the ability to confidently provide a result that informs fluoroquinolone treatment for two-thirds of patients is still clinically relevant and has the potential to enhance antimicrobial stewardship for fluoroquinolones in *M. genitalium*, which is currently lacking in Australia and globally. Additionally, we did not attempt to distinguish the S83R, S83C or the various D87 mutations (e.g. D87N, D87Y and D87H) using either assay. While this may theoretically be possible based on the lower melting temperatures (as shown in Figure 2), we believe trying to interpret these lower temperature curves may potentially undermine confidence in these results, particularly noting that other SNPs (such as G81C, G81S and D82N) may also potentially lower melting temperatures.

In summary, we have developed simple molecular assays that can accurately distinguish *M. genitalium* WT and mutant *parC* sequences. This development is particularly timely, noting there is limited commercial interest in the area, with new evidence showing high associations between WT ParC and fluoroquinolone cure rates^19,20^ and the increasing need to individualize treatment to improve cure and antimicrobial stewardship for this resistant STI. These types of assays have considerable potential to improve resistance-guided therapy approaches and rationalize tests of cure for *M. genitalium* infection.

## Supplementary Material

dkac097_Supplementary_Data

## Supplementary data

Table S1 is available as Supplementary data at *JAC* Online.
